# Maternal immune activation generates anxiety in offspring: A translational meta-analysis

**DOI:** 10.1038/s41398-021-01361-3

**Published:** 2021-04-26

**Authors:** Laiana A. Quagliato, Ursula de Matos, Antonio E. Nardi

**Affiliations:** Laboratory of Panic & Respiration, Institute of Psychiatry, Federal University of Rio de Janeiro, 22270-010 Rio de Janeiro, Brazil

**Keywords:** Epigenetics and behaviour, Predictive markers

## Abstract

Maternal immune activation (MIA) during pregnancy is recognized as an etiological risk factor for various psychiatric disorders, such as schizophrenia, major depressive disorder, and autism. Prenatal immune challenge may serve as a “disease primer” for alteration of the trajectory of fetal brain development that, in combination with other genetic and environmental factors, may ultimately result in the emergence of different psychiatric conditions. However, the association between MIA and an offspring’s chance of developing anxiety disorders is less clear. To evaluate the effect of MIA on offspring anxiety, a systematic review and meta-analysis of the preclinical literature was conducted. We performed a systematic search of the PubMed, Web of Science, PsycINFO, and Cochrane Library electronic databases using the PRISMA and World Health Organization (WHO) methodologies for systematic reviews. Studies that investigated whether MIA during pregnancy could cause anxiety symptoms in rodent offspring were included. Overall, the meta-analysis showed that MIA induced anxiety behavior in offspring. The studies provide strong evidence that prenatal immune activation impacts specific molecular targets and synapse formation and function and induces an imbalance in neurotransmission that could be related to the generation of anxiety in offspring. Future research should further explore the role of MIA in anxiety endophenotypes. According to this meta-analysis, MIA plays an important role in the pathophysiological mechanisms of anxiety disorders and is a promising therapeutic target.

## Introduction

Maternal immune activation (MIA) during pregnancy is recognized as an etiological risk factor for various psychiatric and neurological disorders in offspring^1^. The highly orchestrated processes of neural development start with the proliferation and migration of glia and neurons followed by programmed cell death, the formation of synapses, myelination, and the establishment of neuronal circuits^2^. Therefore, inflammation in the mother during pregnancy can affect several vulnerable aspects of fetal brain development^3^. This disturbance may contribute to a wide spectrum of neuronal dysfunction and behavioral phenotypes in the progeny^3^.

Considering that MIA might lead to altered behavior in offspring, an alternative model has emerged as an explanation for the etiology of psychiatric disorders: the two-hit model^4–6^. In this model, two “hits” are required for the emergence of disorders in offspring: a first “hit”, which occurs during prenatal life (such as MIA) and disrupts the offspring’s central nervous system (CNS) development, thereby increasing the vulnerability to a second “hit”, which might occur later in life and leads to the onset of the disorder^7^. In many cases, the second “hit” could be an environmental factor such as psychological stress^7,8^.

One of the common animal models used to study the two-hit model of psychiatric disorders is induced by MIA, which is achieved by exposing a dam to polyinosinic:polycytidylic acid (PolyI:C) or lipopolysaccharide (LPS), which mimic viral or bacterial insult, respectively, during pregnancy^7^. Both of these agents stimulate the production of many endogenous proinflammatory cytokines, including interleukin (IL)-1β, IL-6, and TNFα, which, along with other factors, recruit and stimulate the production of immune cells^9^. These modifications were shown to induce behavioral deficits in offspring and elicit changes in gene expression in their brains^10^.

**Fig. 1:**
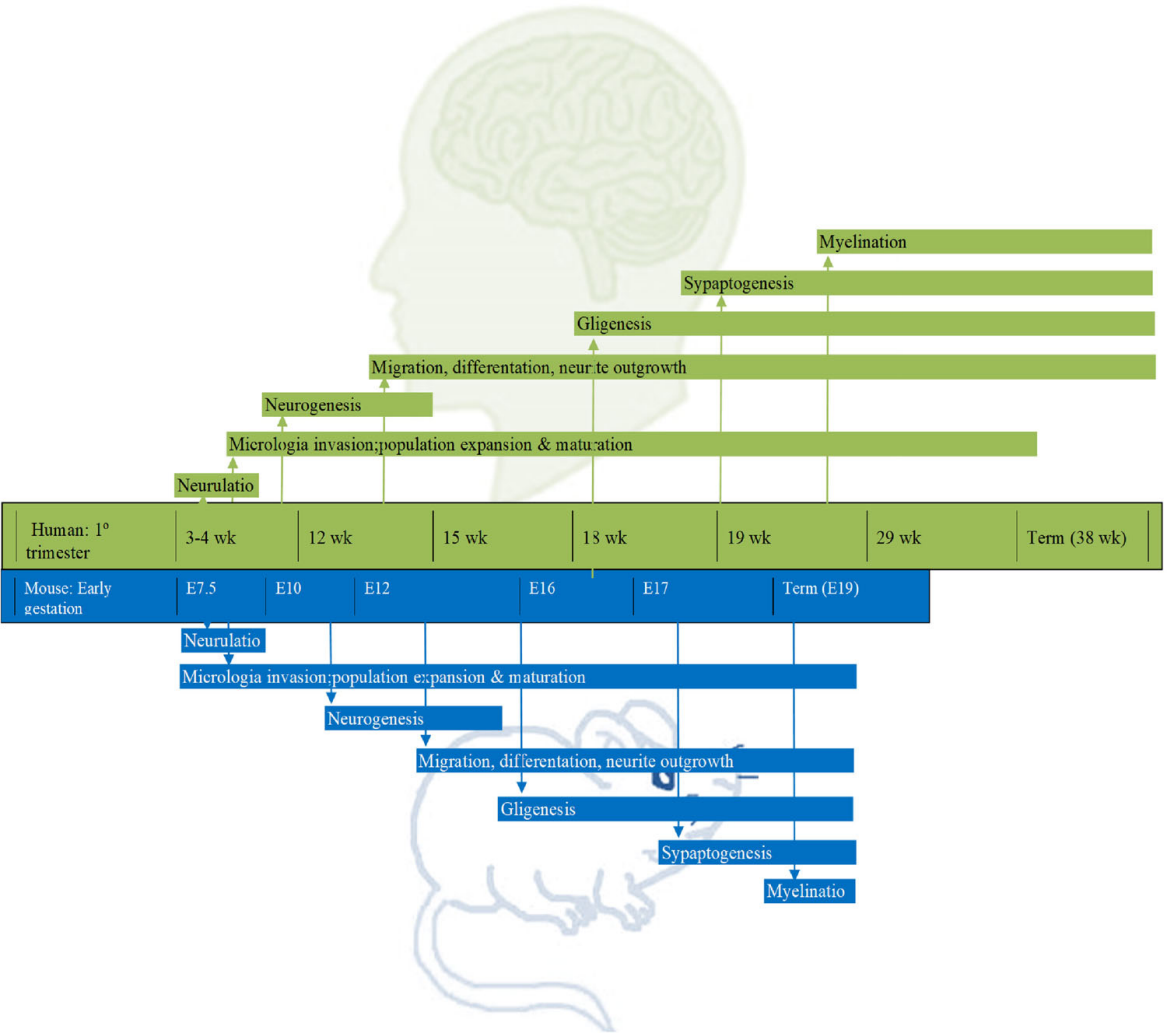
Prenatal forebrain development in rodents and humans and the different timescales. Similarities between the time course of key neurodevelopmental processes in humans and rodents. wk week, E embryonic day.

Preclinical studies are of greatest translational value when they focus on clinically relevant mechanisms and behaviors^11^. Anxiety symptoms, for instance, might be evaluated in animals by different tests, namely, the elevated plus-maze and escape behavior test^12^. The elevated plus-maze is widely used to analyze the behavior of rodents, and it has been validated as an assessment of anxiety in preclinical studies. Briefly, a rat or mouse is placed at the junction of the four arms of the maze facing an open arm, and the duration of time spent in each arm within 5 min is recorded simultaneously by a video-tracking system and observer. An increase in the duration of time spent in the open arm reflects anti-anxiety behavior^12^. The most common animal species used in biological psychiatry are mice and rats^13^, which adequately recapitulate many features of human prenatal forebrain development (e.g., neurulation, neural differentiation, proliferation, and migration) although on different timescales^13^ (Fig. 1). MIA in rodents can indirectly influence postnatal developmental processes through early impacts^13^.

The emerging consensus among leaders in the field is that prenatal inflammation may be relevant to a number of CNS diseases, and restricting interpretation to any given human disorder may limit the utility and relevance of the MIA model^14–16^. Prenatal immune challenge may serve as a “disease primer” for alteration of the trajectory of fetal brain development that, in combination with other genetic and environmental factors, may ultimately result in the emergence of different CNS pathologies^17–19^. Consistently, it has been shown that there is an association between MIA and an enhanced risk of schizophrenia^20–22^, autism^17,23,24^, and depression^11,25,26^ in adult offspring. Nevertheless, the association between MIA and an offspring’s chance of developing anxiety disorders is less clear. Therefore, a systematic review and meta-analysis of the preclinical literature investigating the effects of MIA on offspring anxiety symptoms was conducted. In this framework, we aimed to assess whether prenatal immune activation impacts anxiety behaviors in offspring.

## Methods

This systematic review was performed according to the Preferred Reporting Items for Systematic Reviews and Meta-analyses (PRISMA) guidelines^27^ and World Health Organization (WHO) Review Protocol Template Guidelines, where applicable, as presented in Supplementary Materials Section 1.

### Search strategy

Databases including PubMed, Web of Science, PsycINFO, and the Cochrane Library were searched from inception to 25 March 2021. No language or date restrictions were applied. To avoid publication bias, non-English-language studies and gray literature (for example, conference abstracts) were included. The flowchart of the literature search is presented in Supplementary Fig. 1. A broad but highly structured search strategy based on the PICOS framework was used^28^. Studies were included if they met the following criteria regardless of design: (a) pregnant animals were used for the maternal group, and the offspring were evaluated; (b) during pregnancy, MIA was induced in or a placebo was administered to the maternal group; and (c) the anxiety-related behavioral phenotypes (specifically those assessed by preclinical tests of anxiety disorders^12^, such as the elevated plus-maze, elevated T-maze, and/or analysis of escape behavior induced by electrical/chemical stimulation of the periaqueductal gray matter) of offspring were evaluated. A full list of terms used for the search strategy can be found in Supplementary Materials Section 2.

### Data collection process

Two authors (L.A.Q. and U.M.) reviewed the titles and abstracts and excluded studies clearly unrelated to this review. The search results were evaluated in three consecutive stages. First, the titles and abstracts of all electronic articles were screened. The full test of the articles that presumably met the inclusion criteria were accessed. Finally, all studies reporting the outcome of interest were included in this review. If multiple publications were identified to have studied the same cohort, the most recent or most complete publication was used for data extraction.

### Data extraction and quality assessment

The following data were extracted from each included study independently by two authors in duplicate: title, name of the first author, year of publication, MIA method, time window of MIA, sample size, percentage of animals of each sex, group comparison, age at sample collection, and outcomes. Two independent reviewers (L.A.Q. and U.M.) assessed the quality of the studies using the Syrcles Bias Tool^29^ (Table S1).

### Statistical analysis

Random-effects pairwise meta-analyses using the DerSimonian and Laird random-effects model were conducted. The main outcome measure was the presence or absence of validated preclinical anxiety behavior in the offspring of dams exposed to MIA versus those not exposed to MIA, the results were considered statistically significant at the *α* = 0.05 level. We used the standardized mean difference, which was obtained by calculating the mean (SD) and sample size (*n*) of the MIA offspring group versus the non-MIA offspring group, as the summary statistic. When SDs were not available, we estimated them based on the other statistical parameters reported in the study or requested them from the authors. The *I*^2^ statistic was used to quantify heterogeneity, with an *I*^2^ value of 0% indicating no observed heterogeneity and larger values indicating increased heterogeneity. All statistical analyses were performed using RevMan 5.3 (RevMan; The Cochrane Collaboration, Oxford, UK). We assessed publication bias using funnel plot techniques, Begg’s rank test, and Egger’s regression test, as appropriate, given the known limitations of these methods.

## Results

The literature search identified 110 potentially relevant articles for initial screening. Duplications (*n* = 38) were identified and excluded by manual screening of the titles. Forty studies were excluded from the first assessment of titles and abstracts since they did not meet the selection criteria mentioned above. In all, 32 full texts, of which 5 met the inclusion criteria for our meta-analysis, were reviewed (Supplementary Fig. 1). All the studies evaluated adult progeny and used the elevated plus-maze test to assess anxiety behavior.

**Fig. 2:**
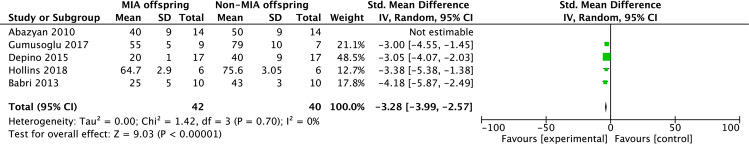
Forest plot. Forest plot. Comparator: offspring exposed to MIA × offspring not exposed to MIA. Outcome: time spent in the open arms in the elevated plus-maze.

Publications included in the meta-analysis used a total of 82 animals and investigated the time spent in the open arm by offspring in the elevated plus-maze test (Table 1)^30–34^. Adult offspring were evaluated after experiments that may have induced MIA in their mothers were performed. The main outcome measure of this meta-analysis was how long the offspring of dams exposed to MIA and those of dams not exposed to MIA spent in the open arms in the elevated plus-maze test. The overall effect size of a reduced time spent in the open arms in the elevated plus-maze test as a result of MIA was −3.28 [95% CI −3.99 to −2.57], and no substantial heterogeneity was observed (*I*^2^ = 0%, *p* = 0.70) (Fig. 2). Publication bias was not assessed, as there was an inadequate number of included studies to properly assess a funnel plot or to perform more advanced regression-based assessments.

**Table 1:** Studies included in the systematic review and meta-analysis.

Study	Species	MIA method	Time of MIA	*N* (MIA)	*N* (controls)	Test	Neuropathological outcomes in adulthood
Abazyan et al. (2010)	Double transgenic mice	Poly I:C	GD9	14	14	EPM	<Serotonin levels
Depino et al. (2015)	C57BL/6J mice	LPS	GD9	17	17	EPM	<Serotonin and noradrenaline levels
Hollins et al. (2018)	Wistar rats	Poly I:C	GD10	6	6	EPM	<Serotonin transporter levels
Gumusoglu et al. (2017)	Mice	Poly I:C	GD12	9	7	EPM	<GABA levels
Babri et al. (2013)	NMRI and CB7BL mice	LPS	GD17	10	10	EPM	>Inflammation in hippocampal tissue

*GD* gestational day, *EPM* elevated plus-maze.

## Discussion

Our meta-analysis demonstrated that progeny exposed to inflammation in utero spent less time in the open arms in the elevated plus-maze test than animals that were not exposed to an inflammatory stimulus during pregnancy. In this test, a shorter time spent in the open arms indicates greater anxiety. Therefore, our meta-analysis showed that inflammation during pregnancy generates anxiety symptoms in adult offspring.

Mechanistically, prenatal exposure to inflammation might contribute to abnormalities in the CNS, including GABAergic delay, attenuated serotonin and noradrenaline neurotransmission, reduced growth of the lateral ventricles, decreased amygdala, and periaqueductal gray matter volumes, decreased density of dendritic spines on granule cells in the hippocampus, and increased microglial reactivity^30,32,33^. However, the specific molecular mechanisms by which MIA contributes to anxiety in offspring have yet to be elucidated.

Alterations in neurotransmission are thought to be central to many psychiatric disorders, including anxiety^35,36^. To date, much of our knowledge is related to the role of neurotransmitter systems in the adult brain in anxiety. Nevertheless, evidence has shown that catecholamines play an important role in neurodevelopment. The catecholamines serotonin and noradrenaline are involved in neural crest stem cell migration and proliferation^37^. Serotonin can regulate the formation of neuronal microcircuits affecting Reelin secretion. Reelin is a protein involved in neuronal migration and positioning during development^38^. Catecholamines are also critical for neuronal cell survival, growth and differentiation as well as synaptogenesis^37,38^. MIA in the first trimester of pregnancy reduces catecholamine concentrations, affecting neurodevelopment.

MIA in the first trimester also delays the GABA switch, which might be related to the fact that an inflammatory environment promotes a reduction in K^+^–Cl^−^ cotransporter 2 (KCC2) transcription^39^. KCC2 activity maintains a low intracellular Cl^−^ concentration, a prerequisite for effective GABA/Gly-mediated inhibition in the nervous system^39^. Since MIA promotes elevation of intracellular chloride concentrations in the offspring brain, GABA remains excitatory in developmental time windows when it is normally inhibitory^39,40^. This process renders neuronal networks hyperexcitable^39,41^.

Genetic alterations in the KCC2 gene have been reported to confer increased anxiety susceptibility^42^. However, the ability of an environmental stimulus, such as MIA, to modify KCC2 expression and lead to an anxious phenotype is not well established in the literature. Of note, a defect in the depolarizing-to-hyperpolarizing switch, which is responsible for excitatory/inhibitory imbalance, has been identified as a key pathophysiological mechanism in several neurodevelopmental disorders, such as autism and schizophrenia^42^, which are typically associated with MIA^43,44^. The delay in the excitatory-to-inhibitory switch is intrinsically maintained in neurons isolated from the brain and maintained in primary cultures independent of the brain environment due to epigenetic mechanisms that alter neuron developmental trajectories^45^. This is consistent with the induction of behavioral alterations by MIA in the first- generation and second-generation offspring of immune-challenged ancestors, demonstrating the transgenerational nongenetic inheritance of pathological traits^45^.

**Fig. 3:**
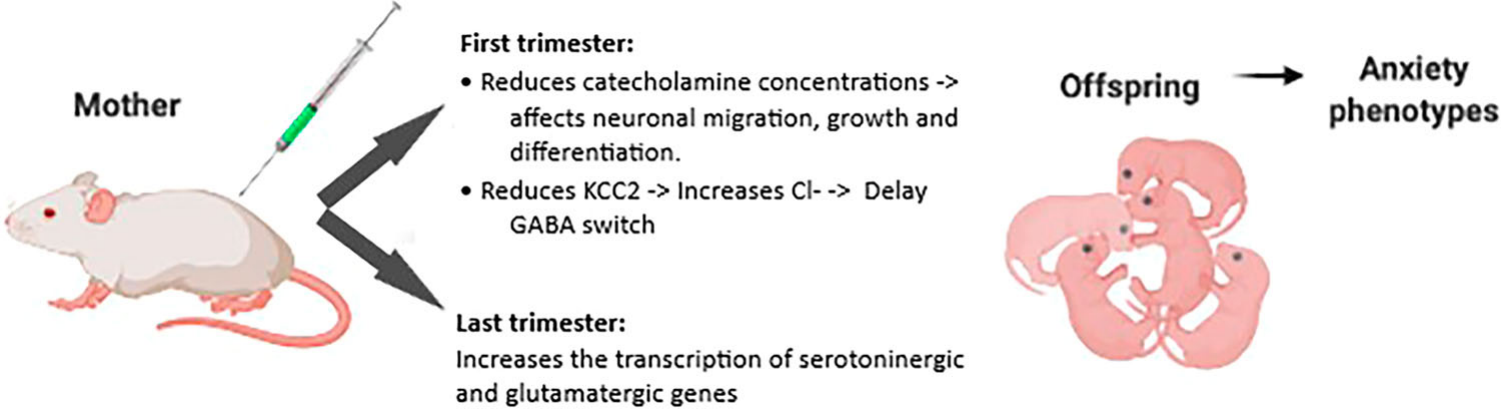
Immunological stimulation of a gestating dam with PolyI:C or LPS results in a variety of molecular alterations, neuronal dysfunction, and anxiety phenotypes in the offspring. Maternal immune activation results in neurotransmission alterations and affects neuronal migration, growth, and differentiation. PolyI:C polyinosinic:polycytidylic acid, LPS lipopolysaccharide, KCC2 potassium chloride cotransporter, Cl chlorideion, GABA gamma-aminobutyric acid.

In the last trimester of gestation, MIA increases the transcription of serotoninergic and glutamatergic genes, which could also contribute to an excitatory–inhibitory imbalance^37^ (Fig. 3). Together, these studies suggest that optimal levels of serotonin, noradrenaline, and GABA must be maintained during development and that any deviations from these optimal levels in either direction can lead to long-lasting behavioral deficits.

Plasticity during the perinatal period is essential for the developing brain to adapt to a changing environment but provides the opportunity for external factors to derail neuronal circuits and lead to maladaptive behaviors^46,47^. This perinatal time window is a critical period when serotonergic, noradrenaline, glutamatergic and GABAergic activity can shape the development of neuronal circuitry and specifically emotional neurocircuits^37,48^.

Our review has several limitations. MIA paradigms are heterogeneous; consequently, the variation in experimental parameters could have an effect on the phenotypes observed^16,49–51^. Additional factors such as housing or the mouse strain may also influence experimental outcomes^34,52,53^. Therefore, experimental guidelines for MIA should be followed so that we can better understand the specific long-lasting effects of prenatal LPS or PolyI:C exposure on offspring physiology and behavior.

Findings from translational rodent models of anxiety disorders could provide information that may contribute to our understanding of the pathophysiology of this disorder in humans. According to this meta-analysis, MIA plays an important role in the pathophysiological mechanisms of anxiety disorders. While there is a clear need for more studies addressing these issues in primates and cross-validation between species, MIA could be a promising therapeutic target for anxiety disorders. Future studies should focus on investigating the interactions between inflammation and genetic factors as well as with other environmental factors, such as diet and drug exposure, which may be important mediators of the neural consequences of maternal infection. Studies aimed at translating findings from animal models to humans may facilitate the identification of new risk factors, mechanistic pathways, and interacting genetic mutations related to MIA and anxiety disorders. Moreover, further studies may better elucidate the relationships between MIA and structural and functional brain phenotypes associated with anxiety disorders.

## Supplementary information

Supplemental Material
