# Supplementary material for: Maternal immune activation generates anxiety in offspring: A translational meta-analysis
Source: Transl Psychiatry. 2021 Apr 26;11:245. doi: 10.1038/s41398-021-01361-3 (PMC8076195; doi:10.1038/s41398-021-01361-3)

**Appendix 1: Search Protocol**

# Systematic Search Protocol

*Written based on the WHO Review Protocol Template, 2011*

Title: **Does maternal immune activation generate anxiety in offspring? A translational systematic review**

1. Background: Maternal immune activation (MIA) during pregnancy is recognized as an etiological risk factor for various psychiatric disorders. However, the association between MIA and the offspring’s chances for developing anxiety disorders is less clear.
2. Objective: This review aims to examine the effect MIA in offspring anxiety.

1. Review Question (Population Intervention Comparison Outcome)

Population: any animal model for genitors and included an offspring

Intervention: during pregnancy was evocated MIA from genitors’ group

Comparison: a comparison group was preferable

Outcome: offspring with anxiety behavior phenotypes were reported

Evidence Gathering and Study Selection: Web of Science, Psycinfo, Cochrane Library and Pubmed databases will be searched. Full search terms for each database and results found per database are listed in the Appendix 2.

Reference searches: Bibliographies of papers deemed eligible for this review will be hand searched to identify any additional eligible references, which will then be screened for title, abstract or full text as appropriate.

1. Eligibility Criteria

The results of these searches will be combined and deduped using Endnote. They will then be screened for title and abstract, and then full text using the following eligibility criteria.

1. Type of study included: any
2. Types of participants: any animal model for genitors, which included an offspring
3. Types of outcome measures: offspring with anxiety behavior phenotypes were reported

1. Exclusion Criteria

Reviews, studies that did not report original data, studies without an anxiety animal model, and in vitro studies will be excluded.

1. Data extraction

Data extracted will include: Title, name of the first author, year of publication, MIA method, time window of MIA method, sample size, sex percentage, group comparison, age at sample collection, modifications in neurocircuitry. The final decision on what to include in the published tables will be made by the systematic review author team based on importance and variability within the studies.

1. Data Synthesis

Narrative synthesis is planned. Statistical synthesis is not expected to be possible as there is wide variability in the types of measures, types of outcomes and brain regions investigated, but will be considered if feasible given the data.

1. Dissemination

A manuscript will be prepared for submission to a peer reviewed journal in the neuroscience field.

**Appendix 2: Search terms**

"maternal immune"OR "two hit"OR "dual hit" AND Brain*OR neurodevelopment* OR fetus OR embrio*OR "brain maturation"OR "brain growth"OR "brain morphology"OR neuro*OR psychopathology OR "neuropsychiatric disorders"OR synaptic OR synapsis OR behavior AND anxiety

**Appendix 3: Custom data extraction form used for included studies**

| **Study ID:** | | |  |
| --- | --- | --- | --- |
| **Coder initials:** | | |  |
| **Date started:** | | |  |
| **Date completed:** | | |  |
| **Title of the paper:** | | |  |
| **Year of publication:** | | |  |
| **Journal:** | | |  |
| **Location (country):** | | |  |
| **Study design** | | |  |
| **1. Randomised controlled trial** | | |  |
| **2. Controlled trial / pseudo-randomised / quasi-experimental** | | |  |
| **3. Cohort** | | |  |
| **4. Clinical audit** | | |  |
| **5. Case-control** | | |  |
| **6. Case series** | | |  |
| **7. Case report** | | |  |
| **8. Other design (specify)** |  | |  |
| **Population description (Strain):** |  | |  |
| **Sample size:** |  | |  |
| **Sex of animals** |  | |  |
| ***n* (males)** |  | |  |
| ***n* (females)** |  | |  |
| **Age of animals** |  | |  |
| **Mean** |  | |  |
| **Range** |  | |  |
| **Primary aim of study:** |  | |  |
| **Intervention / level of exposure** | **Prescription** | | **Reported** |
| **Number of participants** |  |  |  |
| **Duration of treatment** |  |  |  |
| **How did the study define the intervention?** |  |  |  |
| **How did the study deliver the intervention?** |  |  |  |
| **Time of MIA method** |  |  |  |
| **Type of comparison** | | |  |
| **Comparator** | **Prescription** | | **Reported** |
| **Number of participants** |  |  |  |
| **Duration of treatment** |  |  |  |
| **How did the study define the comparator?** |  |  |  |
| **How did the study deliver the comparator?** |  |  |  |
| **Outcome** |  |  |  |
| **Is outcome tool validated?** |  |  |  |
| **Time points measured** |  |  |  |
| **Between Groups** |  |  |  |
| **Effect size (difference between groups)** |  |  |  |
| **Effect size (%) (if different units)** |  |  |  |
| **Level of significance (P-value or CI)** |  |  |  |
| **Sponsorship / funding (verbatim):** |  |  |  |
| **Authors conflicts of interest** |  |  |  |

**Appendix 4: Supplementary Figure 1. PRISMA Flow Diagram.**

Studies included in quantitative synthesis (meta-analysis)
(n = 5 )

Records excluded
(n = 40 )

Records screened
(n = 72 )

Records after duplicates removed
(n = 72 )

Additional records identified through other sources
(n = 0 )

Records identified through database searching
(n = 110)

## Identification

## Screening

Full-text articles excluded, with reasons
(n = 27 )

N= 20, animal studies with non-validated models for anxiety disorders.

N=3, studies that didn’t evaluated MIA during pregnancy

N=3, narrative reviews

N=1, chapter

Full-text articles assessed for eligibility
(n = 32 )

## Eligibility

Studies included in qualitative synthesis
(n =5)

## Included

**Appendix 5: Supplementary table 1. Quality score.**


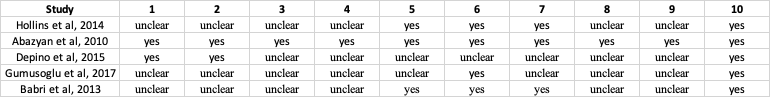

Supplement: Supplementary file 1 — Supplemental Material [file 41398_2021_1361_MOESM1_ESM.docx]
